# Pneumococcal meningitis and endocarditis in an infant: possible improved survival with factor V Leiden mutation

**DOI:** 10.1007/s00431-017-2973-1

**Published:** 2017-08-12

**Authors:** Sitikant Mohapatra, Assaf Doulah, Elspeth Brown

**Affiliations:** 1 Paediatric Intensive Care Unit, Leeds Teaching Hospitals NHS Trust, Leeds, LS1 3EX UK; 2 Paediatric Cardiology, Leeds Teaching Hospitals, Leeds, LS1 3EX UK

**Keywords:** Endocarditis, Factor V Leiden, Meningitis, Pneumococcal

## Abstract

Streptococcus pneumoniae infections continue to remain associated with high morbidity and mortality. Although the incidence of invasive meningeal and/or lung disease are not uncommon, Streptococcus pneumoniae endocarditis is rare especially in healthy pediatric population. New studies have suggested a strong association between factor V leiden (FVL) mutation and favorable outcomes in critically ill children. A healthy 10 month old presented with sepsis and meningeal signs, was later confirmed to have Streptococcus pneumoniae meningitis and endocarditis. She was found to have factor V leiden mutation and made a complete recovery despite initial complications.

*Conclusion*: Presence of factor V leiden mutation in critically ill children with severe septicaemia possibly contributes to better outcomes.

**Table:** 

**What is known:** • Mortality and morbidity remain high with invasive pneumococcal disease. • Pneumococcal endocarditis is rare in healthy pediatric population and results in significant morbidity and mortality
**What is new:** • New studies have suggested a strong association between factor V leiden (FVL) mutation and favorable outcomes in critically ill children. • The presence of factor V mutation in children with extensive invasive pneumococcal disease possibly contributes to a better outcome.

## Introduction

Streptococcus pneumoniae endocarditis occurs less commonly in children and accounts for only 3–7% of cases of endocarditis in children [1]. Most cases tend to occur in children with congenital heart disease, those with previous cardiac surgeries, or associated with vascular catheters. Such endocarditis in previously healthy heart is rarely reported. Association of Streptococcus pneumoniae endocarditis with meningitis has been reported in adult literature but such an association in children is rare [2].

Activated factor V or factor Va is an important cofactor for conversion of prothrombin to thrombin which leads to formation of blood clots. FVL is the name given to the specific mutation which leads to the hypercoagulability state with serious clinical consequences. The Leiden mutation leads to elimination of one of the several sites on factor Va that are substrates for endogenous proteases like activated protein C (APC). This results in resistance of factor V to APC, diminished inactivation of factor Va, and preventing the formation of its anticoagulant form FVac. APC resistance of FV Leiden thereby enhances the thrombosis risk of heterozygous and homozygous carriers by 4- to 6-fold and by 30- to 80-fold, respectively. [3].

## Illustrative case

A 10-month-old previously well child with a short history of fever presented to the hospital with abnormal movements and posturing and signs of meningeal irritation. She was hemodynamically compromised and needed a total of 50 mls/kg of fluid for poor perfusion. The abnormal posturing and fits terminated following benzodiazepines, paraldehyde, and loading with phenytoin. In view of low Glasgow coma scale, she was intubated and taken for a computed tomography (CT) scan of the head which revealed bilateral fronto-temporal subdural effusions with mild hydrocephalus. Prior to presentation, she was fit and well and not immunized.

**Fig. 1:**
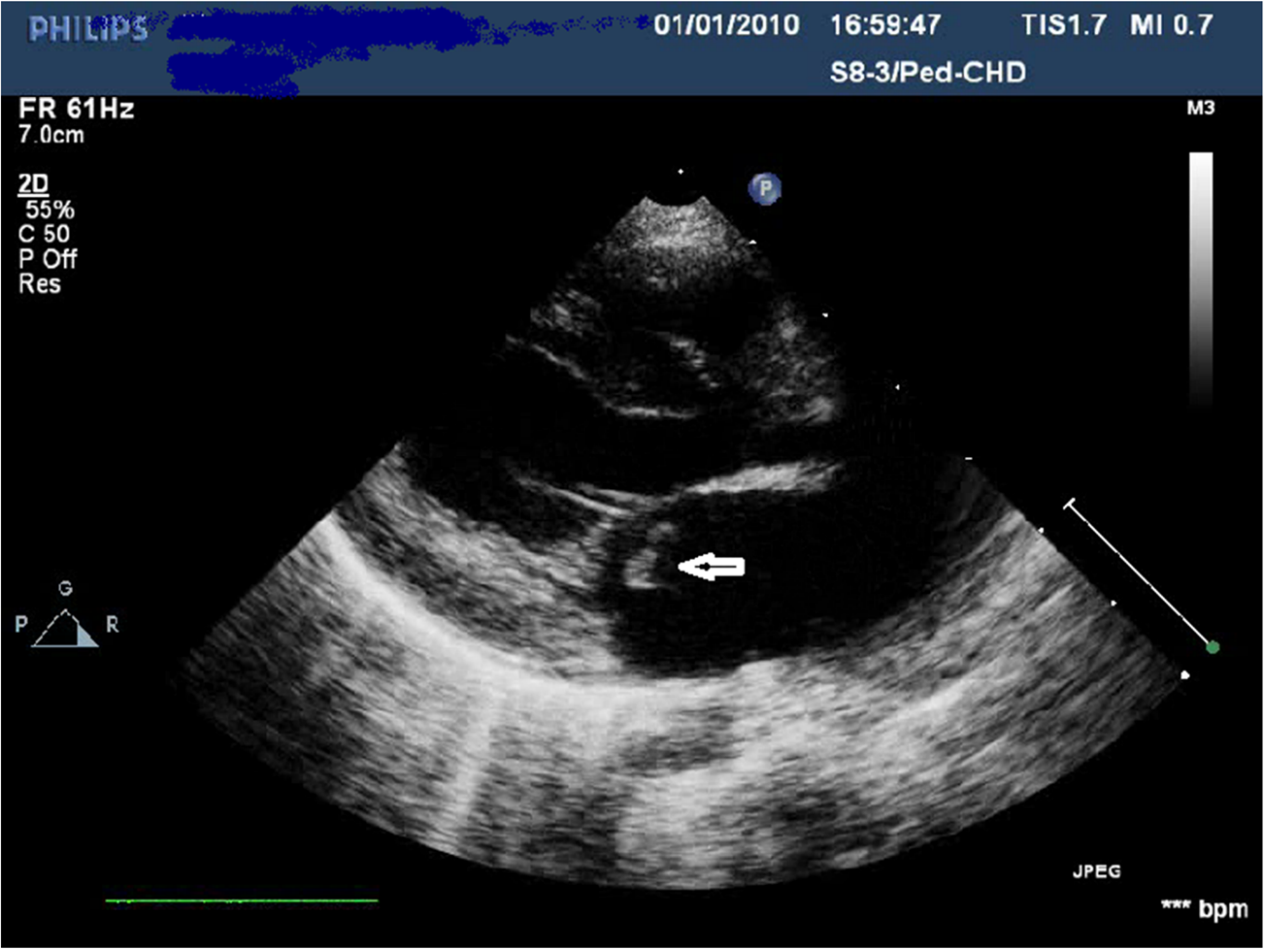
Echo ardiogram of the 10 month old infant showing vegetation attached to the mitral valve

She was transferred to pediatric intensive care unit where she was hemodynamically stable without the need for inotropes. A murmur was noticed at admission which was followed up by an echocardiogram. The echocardiogram revealed normal cardiac anatomy with good ventricular function but two distinct vegetations attached to the mitral valve with moderate mitral valve regurgitation (Fig. 1).

She was initially given broad antibiotic cover with cefotaxime (50 mg/kg/dose IV 6 hourly), clarithromycin (7.5 mg/kg/dose IV 12 hourly), and acyclovir (500 mg/m^2^/dose IV 8 hourly). Blood cultures sent at admission did not reveal any growth and polymerase chain reaction (PCR) results on blood tested negative for herpes simplex virus and meningococcus but positive for Streptococcus pneumoniae. Immunoglobulin M (IgM) for mycoplasma was also negative. In view of the vegetations on the mitral valve, rifampicin (7.5 mg/kg/dose IV 12 hourly) was added to cover for Streptococcus pneumoniae endocarditis. Cerebrospinal fluid after lumbar puncture revealed eight white blood cells (two polymorphs and six lymphocytes), CSF protein 0.46 g/l, and CSF glucose 2.9 mmoles/l and cultures did not reveal any growth after 7 days. However, CSF sample sent for PCR was positive for Streptococcus pneumoniae, as was urine sample for Streptococcus pneumoniae antigen. Ultrasound of the abdomen including both kidneys and spleen were normal. Antibiotics were therefore rationalized and she received a 6-week course of cefotaxime and rifampicin in line with treatment for Streptococcus pneumoniae endocarditis. In addition, as a complimentary therapy, her parents used kinesiology techniques to aid healing.

A repeat CT scan of the head on day 2 revealed shallow subdural effusions. An electroencephalogram showed excessive slow wave activity consistent with sedation or cortical dysfunction with no evidence of non-convulsive status. She developed right facial palsy and weakness of right lower limb on week 2. CT head repeated a week after initiation of treatment showed evolving infarction in the left tempero-parietal area consistent with a middle cerebral artery branch infarct and increase in hydrocephalus which was a new finding. She was anticoagulated and a thrombophilia screen was performed. Protein C, antithrombin III, protein S levels IgG anticardiolipins, and lupus anticoagulant were normal. INR, PT, APTT, and fibrinogen were within normal limits. She was found to be a heterozygous (carrier) for FVL mutation and APC resistance was low (1.6) in line with heterozygous carrier of FVL mutation.

Over the subsequent 3 weeks, her right hemiparesis and facial palsy recovered completely. Repeat echocardiography during follow-up showed mild mitral regurgitation possibly consequent to a perforation in the anterior leaflet of the mitral valve without any signs of left ventricular dysfunction or dilatation of the left heart.

She remained under regular follow-up. Her development remains appropriate for age with no neurological deficit. A formal hearing assessment showed no hearing deficit.

## Discussion

Invasive Streptococcus pneumoniae infections are associated with significant morbidity and mortality in the pediatric population. A recent series reported an overall case fatality rate of 13%. Of the survivors, 63% had neurologic sequelae [4].

In about 8–10% of pediatric cases of infective endocarditis (IE), there is no pre-existing structural heart disease or other identifiable risk factors. These cases usually involve infection of the aortic or mitral valve secondary to *Staphylococcus aureus* bacteremia [1, 4]. Children with congenital or acquired immunodeficiency but without other identifiable risk factors for IE do not seem to be at increased risk for endocarditis compared with the general population. Factors often associated with IE in adults, such as intravenous drug abuse and degenerative heart disease, are not common predisposing factors in children [1]. Cases of Streptococcus pneumoniae IE with healthy native valve, is rarely reported.

The possibility of existence of multiple foci of such infection, i.e., Streptococcus pneumoniae meningitis and endocarditis is rare especially in previously healthy children. The incidence of pneumococcal endocarditis in children with invasive Streptococcus pneumonia disease is 0.4% [2]. With a lack of classical symptoms of IE and a very high incidence of mortality (11–18%) [2], it is imperative to appropriately treat co-existent IE as inadequate treatment could result in serious complications including death.

A review of 11 children with Streptococcus pneumonia endocarditis between September 1993 and February 2003 revealed that 10 (91%) had pre-existing structural heart disease [2]. One had meningitis and two had pneumonia and meningitis. Complications following Streptococcus pneumoniae IE are common with 36% needing cardiac surgery during hospitalization [2]. Medical therapy alone sometimes resulted in a high mortality rate that was improved in the group of patients receiving combined medical and surgical interventions. Hence, some have suggested that early surgical intervention might improve survival [5].

FVL mutation is the most common genetic cause of venous thrombosis in Caucasians, present in 4–6% of Caucasian population. Such a high prevalence has prompted studies to assess whether such a mutation provides a survival benefit. The clinical consequence of such a mutation, including its role in situations like sepsis where inflammation is prevalent, still remains controversial [6]. The interaction between the presence of FVL mutation and the outcome of severe infection and sepsis is less clear, given that experimental and clinical studies show divergent results [7]. In PROWESS, 4.1% (*n* = 65) of patients were heterozygous FVL carriers and the 28-day mortality was lower in FVL carriers (13.9%) than in non-FVL (27.9%) patients (*P* = 0.013) [3]. Kerlin et al. reported that heterozygous carriership for the FV Leiden allele was indeed associated with reduced mortality of septic patients enrolled in the placebo arm of the PROWESS sepsis trial and in mouse models of sterile lethal inflammation triggered by infusion of bacterial lipopolysaccharide [6]. Kerschen et al. demonstrated in mice that heterozygous carriers of the FV Leiden allele benefit from a selective survival advantage over homozygous FV Leiden carriers and carriers of the normal FV allele in two distinct modes of infection with human bacterial pathogens: gram-positive *S. aureus* and gram-negative *Y. Pestis* [8]. Prospective collection of FVL status from combining the PROWESS and ENHANCE studies of severe sepsis suggested that compared to non-Leiden carriers, FVL heterozygous carriers may have a slightly decreased risk of developing severe sepsis from infection, do not seem to have increased mortality in severe sepsis, and derive similar benefit and risk profiles from drotrecogin alfa (activated) treatment [9]. The striking magnitude of the FVL mutation survival benefit in the initial PROWESS population, and in mice, suggests that its role as a potent endogenous modifier of the pathogenic pathways engaged in sepsis [9].

## Conclusion

In this previously healthy 10 month old who initially presented with symptoms suggestive of meningitis, a high index of suspicion lead to the diagnosis of endocarditis after a soft cardiac murmur was investigated. She made good recovery after being treated with a 6-week course of appropriate antibiotics. The discovery of FVL mutation was made when she was investigated for possible pro-thrombotic states. Although timely intervention and appropriate management were the most important factors resulting in such a good recovery in this child, we wonder if the presence of FVL mutation could have contributed to such a favorable outcome.

## Abbreviations

APC: ᅟ
CT: ᅟ
FVL: ᅟ
IE: ᅟ
PCR: ᅟ
